# Shaping the future of blood management in metastatic spine tumor surgery: the case for cell-salvaged transfusion with a propensity-matched study

**DOI:** 10.1016/j.xnsj.2026.100862

**Published:** 2026-02-05

**Authors:** Si Jian Hui, Jiong Hao Tan, Shen Liang, Yong Hao Tan, Balamurugan Vellayappan, Namith Rangaswamy, Laranya Kumar, Youheng Ou Yang, James Thomas Patrick Decourcy Hallinan, Naresh Kumar

**Affiliations:** a Department of Orthopaedic Surgery, University Spine Centre, National University Hospital, 1E, Lower Kent Ridge Road, Singapore 119228, Singapore; b NUS Medicine Biostatistics Unit (BSU), National University Health System, 10 Medical Drive, Singapore 117597, Singapore; c Department of Radiation Oncology, National University Hospital, 5 Lower Kent Ridge Rd, Singapore 119074, Singapore; d Royal College of Surgeons of Ireland, Dublin, Ireland; e SingHealth Duke-NUS Spine Centre, Singapore General Hospital, 20 College Road, Academia, Level 4, Singapore 169865, Singapore; f Department of Diagnostic Imaging, National University Hospital, 5 Lower Kent Ridge Rd, Singapore 119074, Singapore

**Keywords:** Metastatic spine tumor surgery, Salvaged blood, Blood transfusion, Allogenic blood, Intraoperative cell salvage

## Abstract

**Background:**

Blood loss is a major perioperative concern in metastatic spine tumor surgery (MSTS). Allogeneic blood transfusion (ABT) remains the standard method of blood replacement but is associated with well-recognised complications. Salvaged blood transfusion (SBT) using intraoperative cell salvage may mitigate many of these risks; however, its oncological safety and long-term outcomes in MSTS remain controversial.

**Methods:**

This was a prospective cohort study of patients who underwent MSTS between 2014 and 2017. Clinical outcomes included overall survival (OS) and tumor progression (TP), assessed using RECIST (version 1.1). A propensity score–matched cohort was generated using relevant predictors of treatment allocation and outcomes of interest to enable comparison between patients receiving SBT and ABT.

**Results:**

A total of 98 patients (mean age 60 years) were included, of whom 33 received SBT, 39 received ABT, and 26 received no blood transfusion. Median estimated blood loss was 400 mL (IQR 200–900 mL), and median blood transfusion volume was 328.5 mL (IQR 0–1042 mL). Propensity score matching yielded 30 patients in the ABT group and 28 in the SBT group. There was no significant difference in overall survival between patients receiving SBT and ABT (p=.250). Importantly, SBT was not associated with an increased risk of 4-year TP (p=.908).

**Conclusions:**

SBT demonstrates comparable long-term survival and TP outcomes to ABT in patients undergoing MSTS, while avoiding the known complications associated with ABT. This study represents the first long-term propensity score–matched analysis of SBT in MSTS, supporting its oncological safety and clinical utility in contemporary spine oncology practice.

## Introduction

Global cancer incidence is projected to exceed 35 million new cases by 2050, up from 20 million in 2020 [1]. Advances in diagnosis and treatment have extended survivorship [2], but consequently, the prevalence of metastatic disease continues to rise [3]. The spine is the most common site of bone metastasis [4]. Metastatic spine tumor surgery (MSTS) remains a key modality for metastatic spinal disease (MSD) in patients with neurological compromise, pathological fracture, spinal instability, or intractable pain [5,6].

With improved systemic therapies and longer survival, the surgical threshold for MSD has lowered, allowing patients with higher comorbidity to be considered [7,8]. Despite these advances, intraoperative blood loss remains a major challenge [9,10]. Average blood loss in open MSTS is about 1.4 L, and 0.7 L for minimally invasive approaches [11]; pooled estimates exceed 2 L [9]. In our institution, the mean loss was 911 mL [10].

Most MSTS patients require transfusion. Allogeneic blood transfusion (ABT) remains standard but consumes scarce resources [12] and is associated with infection, immune-mediated lung injury, delayed wound healing, poorer oncologic outcomes, and possibly tumor promotion [13,14]. Consequently, patient blood management (PBM) programs have been advocated to minimize ABT exposure [15]. One key PBM arm is intraoperative salvaged blood transfusion (SBT) [15].

SBT is well established in deformity and degenerative spine surgery [[16], [17], [18], [19]] and proven safe across gynecologic, urologic, hepatobiliary, gastrointestinal, orthopaedic, trauma, and cardiac operations [20,21]. At the biochemical level, cell salvage with leukocyte-depletion filtration yields negligible viable tumor cells [22,23]. Our group previously reported 2-year prospective outcomes showing comparable overall survival (OS) and tumor progression (TP) between SBT and ABT in MSTS [24].

However, existing meta-analyses cite a lack of high-power comparative data between SBT and ABT [25]. A randomised trial is impractical because nearly 40% of MSTS patients present acutely with neurological deficit [26] and the heterogeneity of primary tumors would require unrealistically large samples. Propensity-score (PS) matching therefore, offers the highest attainable evidence level for comparative safety.

In our study, we aim to evaluate the safety and efficacy of SBT in MSTS on long-term follow-up for all MSTS patients, using group PS-matched analysis. This reduces the bias due to confounding factors for estimating treatment effect between those who have received ABT vs. those who have received SBT during MSTS.

## Materials and methods

A single-institution prospective study was conducted on patients who underwent MSTS between January 2014 and December 2017. All patient data were retrieved from the hospital’s electronic medical records. Spinal metastases were diagnosed by magnetic resonance imaging, computed tomography, and/or nuclear bone scans, and indications for surgery included symptomatic spinal cord compression and/or mechanical instability.

Patients were categorized by transfusion type: no blood transfusion (NBT)**,** ABT**,** or SBT (with or without ABT). Primary outcomes were assessed postoperatively through routine records at 6, 12, 24, 36, and 48 months, or until death.

Collected variables included demographics, tumor histology, neurological status (Frankel, ECOG [27], Karnofsky Performance Scale), and metastatic burden (extraspinal, vertebral, visceral). The modified Tokuhashi score [28] was derived for each case. Operative data comprised surgical approach, number of decompressed and instrumented levels, intraoperative blood loss, transfusion volume, and postoperative complications. Tumors were also subclassified by vascularity [29].

Primary outcomes were OS and TP, defined by RECIST v1.1 [30] as ≥20% increase in measurable lesions, unequivocal progression of nontarget disease, or new metastases. Follow-up imaging with computed tomography and magnetic resonance imaging assessed visceral and skeletal progression. RECIST and Tokuhashi scores were independently verified by two blinded authors, with discrepancies resolved by consensus.

### Patient recruitment and transfusion protocol

Patients could elect to join the SBT group or the control (ABT) group, where allogeneic transfusion would be provided if required. They were counselled on potential risks, including the theoretical concern of tumor dissemination, and informed of studies refuting these risks [22,23]. Those consenting received salvaged blood processed through a leukocyte-depletion filter; additional ABT was given if salvaged volume was inadequate. Patients who declined SBT but required transfusion received ABT, while those not needing transfusion comprised the NBT group. Salvaged blood was processed and returned at the end of surgery. Transfusion triggers were standardized (Hb≤8 g/dL or haemodynamic instability). Patients with significant blood loss or dilutional coagulopathy were managed according to ROTEM findings, receiving fresh frozen plasma or platelets as indicated.

### PS-matched analysis

We implemented PS group matching between the ABT and the SBT group to evaluate the outcomes of OS, TP, and peri‑operative complications in the study cohort. Patients were stratified according to clinical variables such as age, gender, race, primary tumor types, Tokuhashi score, number of vertebral metastasis sites, total blood loss, and type of surgery to reduce the bias due to patient-related and confounding factors between both groups. The derived propensity score was categorized into 9 levels in steps of 0.1 for matching of ABT with SBT.

Generalized Linear Mixed Model accounting for the matching was used to show the differences in patients’ demographic and clinical features between ABT and SBT. Patients’ demographic and clinical characteristics were summarised by mean and standard deviation for continuous variables with approximately normal distribution and median (interquartile range) for skewed distribution. Categorical variables were summarized using frequencies and percentages. Univariate Cox proportional hazards regression model was used to study individual continuous variables. Multivariable Cox proportional hazards regression was subsequently conducted to adjust for potential confounders for relationship between BT type and OS. Adjusted hazard ratios accounting for PS matching were obtained using the Cox regression frailty hared function. The association between transfusion type and TP was explored using competing risks analysis, with death without TP considered as the competing event.

### Statistical methods

Statistical analyses were conducted using STATA/SE 14.0 (StataCorp LP). All statistical tests were two-sided with a significance level set at 5%.

## Results

A total of 98 patients were included in this study. This included 53 (54.1%) males and 45 (45.9%) females, with a mean age of 60 years old at the time of surgery. Overall median blood loss was 400 mL (IQR 200–900 mL), and overall median BT was 620 mL (IQR: 110–1600 mL) for patients receiving BT. The demographics and clinical characteristics of the patients are described (Table 1). 33 (33.7%) patients received SBT, 39 (39.8%) received ABT, and 26 (26.5%) had NBT.

**Table 1:** Demographic and clinical characteristics of 98 patients.

Characteristic	Overall (*n*=98)	Blood transfusion (BT) type			
		No BT (*n*=26)	Allogeneic BT (*n*=39)	Salvaged* (*n*=33)	p*-*value
Age at surgery (y), mean±SD	59.8±15.8	54.4±21.3	59.3±13.7	64.6±11.6	.046
Age at surgery, *n* (%)					.012
<60 y	43 (43.9)	16 (61.5)	19 (48.7)	8 (24.2)	
≥60 y	55 (56.1)	10 (38.5)	20 (51.3)	25 (75.8)	
Gender, *n* (%)					.215
Male	53 (54.1)	15 (57.7)	17 (43.6)	21 (63.6)	
Female	45 (45.9)	11 (42.3)	22 (56.4)	12 (36.4)	
Race, *n* (%)					.817
Chinese	63 (64.3)	16 (61.5)	23 (59.0)	24 (72.7)	
Malay	20 (20.4)	6 (23.1)	8 (20.5)	6 (18.2)	
Indian	3 (3.1)	1 (3.8)	2 (5.1)	0 (0.0)	
Others	12 (12.2)	3 (11.5)	6 (15.4)	3 (9.1)	
Total blood loss (mL), median (IQR)	400 (200–900)	200 (50–387.5)	400 (250–925)	700 (390–1450)	.052
Total blood transfusion† (mL), median (IQR) Surgery type, *n* (%)	328.5 (0–1042)	0 (0–0)	500 (0–1081)	705 (1507–2150)	.412, .333
MIS	36 (36.7)	12 (46.2)	16 (41.0)	8 (24.2)	
Posterior open	52 (53.1)	13 (50.0)	19 (48.7)	20 (60.6)	
Corpectomy	10 (10.2)	1 (3.8)	4 (10.3)	5 (15.2)	
Number of instrumentations, *n* (%)					.200
<4	12 (12.2)	5 (19.2)	6 (15.4)	1 (3.0)	
=4	37 (37.8)	11 (42.3)	15 (38.5)	11 (33.3)	
>4	49 (50.0)	10 (38.5)	18 (46.1)	21 (63.7)	
Number of decompressions, *n* (%)					.102
0	25 (25.5)	10 (38.5)	11 (28.2)	4 (12.2)	
1	33 (33.7)	10 (38.5)	11 (28.2)	12 (36.4)	
>1	40 (40.8)	6 (23.0)	17 (43.6)	17 (51.4)	
Total Tokuhashi score, mean±SD	7.08±2.65	6.92±2.71	7.33±2.72	6.91±2.59	.751

SD, standard deviation; IQR, interquartile range.

⁎ Salvaged with or without allogenic BT.

† Overall median total blood transfusion was 620 mL (IQR: 110–1600 mL) for patients receiving BT.

A total of 72 patients (33 SBT and 39 ABT) were used to derive the PS-matched cohort of 28 SBT patients and 30 ABT patients (Table 2). The demographics and clinical characteristics of the patients are described (Table 3). The primary tumor subgroups based on Tokuhashi scoring for both PS-matched ABT and SBT groups were also analyzed (Table 4). Both the PS-matched SBT and ABT groups had nonsignificant differences in the primary tumor subgroups.

**Table 2:** Matching distribution of SBT vs. ABT by propensity score (PS) matching categories.

Propensity categories	ABT (*n*=30)			SBT (*n*=28)		
		Dead	Disease progression		Dead	Disease progression
1	3	2	1	2	2	1
2	3	2	2	3	2	1
3	3	2	0	3	1	0
4	3	3	0	3	2	1
5	3	2	0	3	1	1
6	3	2	1	3	1	0
7	3	2	0	3	1	0
8	3	2	0	3	1	1
9	6	4	0	5	3	2

**Table 3:** Demographic and clinical characteristics of 58 patients (propensity-matched).

Characteristic	Overall (*n*=58)	ABT (*n*=30)	SBT (*n*=28)	p-value
Age at surgery (y), mean±SD	51.3±13.7	58.5±14.4	64.3±12.3	.110
Age at surgery, *n* (%)				.035
<60 y	23 (39.7)	16 (53.3)	7 (25.0)	
≥60 y	35 (60.3)	14 (46.7)	21 (75.0)	
Gender, *n* (%)				.124
Male	31 (53.4)	13 (43.3)	18 (54.3)	
Female	27 (46.6)	17 (56.7)	12 (45.7)	
Race, *n* (%)				.360
Chinese	36 (62.1)	16 (53.3)	20 (71.4)	
Malay	13 (22.4)	8 (26.7)	5 (17.0)	
Indian	2 (3.4)	2 (6.7)	0 (0.0)	
Others	7 (12.1)	4 (13.3)	3 (10.7)	
General condition, *n* (%)				.174
Poor	12 (20.7)	9 (30.0)	3 (10.7)	
Moderate	17 (29.3)	7 (23.3)	10 (35.7)	
Good	29 (50.0)	14 (46.7)	15 (53.6)	
No. of extra spinal met, *n* (%)				.075
≥3 lesions	25 (43.1)	9 (30.0)	16 (57.1)	
1–2 lesions	13 (22.4)	7 (23.3)	6 (21.4)	
0 lesion	20 (34.5)	14 (46.7)	6 (21.4)	
No. of vertebral body mets, *n* (%)				.806
≥3 lesions	44 (75.9)	22 (73.3)	22 (78.6)	
2 lesions	6 (10.3)	3 (10.0)	3 (10.7)	
1 lesion	8 (13.8)	5 (16.7)	3 (10.7)	
Mets to major internal organs, *n* (%)				.865
Nonremovable	22 (37.9)	12 (40.0)	10 (35.7)	
Removable	7 (12.1)	3 (10.0)	4 (14.3)	
None	29 (50.0)	15 (50.0)	14 (50.0)	
Surgery type, *n* (%)				.090
MIS	16 (27.6)	12 (40.0)	4 (14.3)	
Posterior open	33 (56.9)	14 (46.7)	19 (67.9)	
Corpectomy	9 (15.5)	4 (13.30	5 (17.8)	
Number of instrumentation, *n* (%)				.072
<4	7 (12.1)	6 (20.0)	1 (3.6)	
=4	25 (43.1)	14 (46.7)	11 (39.3)	
>4	26 (44.8)	10 (33.3)	16 (57.1)	
Number of decompressions, *n* (%)				.142
0	10 (17.2)	8 (26.7)	2 (7.2)	
1	19 (32.8)	9 (30.0)	10 (35.7)	
>1	29 (50.0)	13 (43.3)	16 (57.1)	
Total Tokuhashi score), mean±SD	7.29±2.59	7.60±2.67	6.96±2.50	.355
Total blood loss (mL), median (IQR)	700 (300–1325)	550 (275–1000)	757.5 (400–1625)	.087
Total blood transfusion (mL), median (IQR)	900 (500–2000)	900 (500–1500)	960 (262.5–2200)	.595
Median survival time (mo)	11.5 (5.3–18.9)	7.1 (4.8–9.5)	18.2 (9.4–27)	.186
Median disease progression (mo)	14.6 (3.5–36.4)	21.4 (0.1–44.8)	6.6 (4.7–8.5)	.908

SD, standard deviation; IQR, interquartile range.

**Table 4:** Primary tumor-type distribution of 58 patients (as per Tokuhashi score).

Primary tumor type	*N* (%)	ABT (*n*=30)	SBT (*n*=28)
Lung osteosarcoma, stomach, bladder, esophagus, pancreas	16 (27.6)	8 (26.7)	8 (28.6)
Liver, gallbladder, unidentified	6 (10.3)	1 (3.3)	5 (17.9)
Rectum	4 (6.9)	2 (6.7)	2 (7.1)
Thyroid, prostate, breast, carcinoid	10 (17.2)	6 (20.0)	4 (14.3)
Kidney, uterus	6 (10.3)	5 (16.7)	1 (3.6)
Other	16 (27.6)	8 (26.7)	8 (28.6)

p=.340.

Comparison of total blood loss between ABT and SBT PS-matched groups revealed no significant difference (550 vs. 757.5 mL; p=.087). Pairwise comparison between ABT and SBT also showed no significant difference in the total amount of blood transfused (900 vs. 960 mL; p=.595).

### Overall survival

**Fig. 1:**
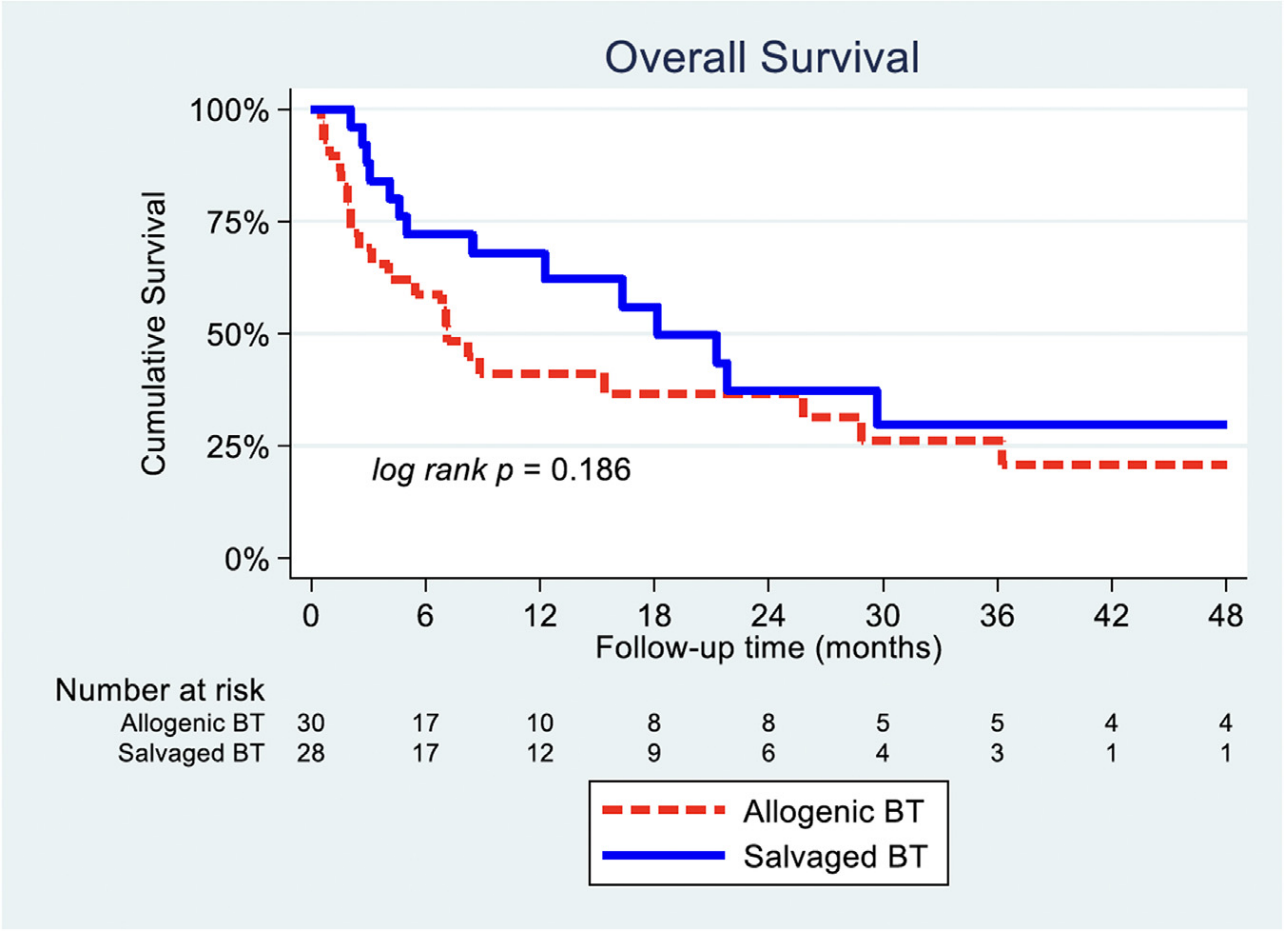
Overall survival: SBT vs. ABT Kaplan–Meier analysis.

**Fig. 2:**
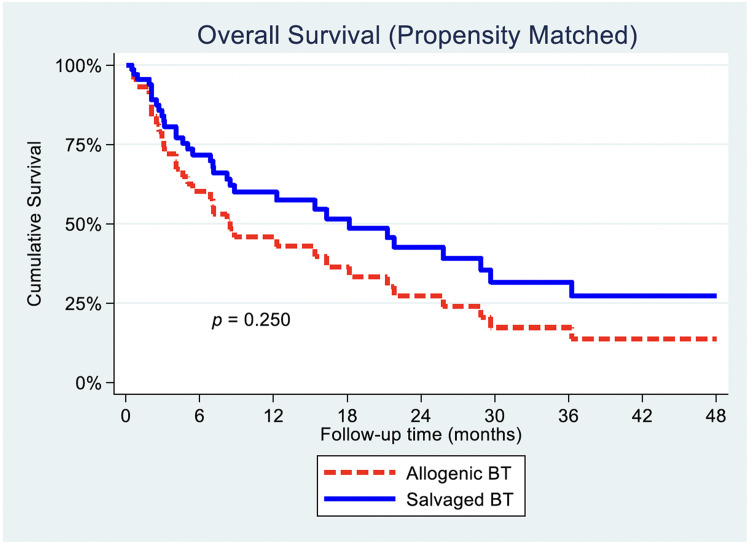
Overall survival: SBT vs. ABT propensity-matched.

OS was comparable between the SBT and ABT group, represented by our adjusted survival curve (Fig. 1). The crude Kaplan–Meier comparison on OS between ABT and SBT demonstrated that median survival time was 7.1 months for ABT (95% CI 4.8–9.5) and 18.2 months for SBT (95% CI 9.4–27.0) (p=.186, unadjusted HR=0.64, 95% CI 0.32–1.3) (Fig. 1). When taking into account for PS matching, there were no statistically significant difference for PS-matched SBT group as compared to PS-matched ABT on our adjusted survival curve (p=.250, HR=0.66, 95% CI 0.32–1.34) (Table 5, Fig. 2).

**Table 5:** Relative risk regression for overall survival and disease progression between matched ABT and SBT groups if SBT was used.

Characteristic	HR (95% CI)	p-value
4-y overall survival	0.66 (0.32–1.34)	.250
4-y disease progression	2.29 (0.8–7.0)	.147

### Disease progression

**Fig. 3:**
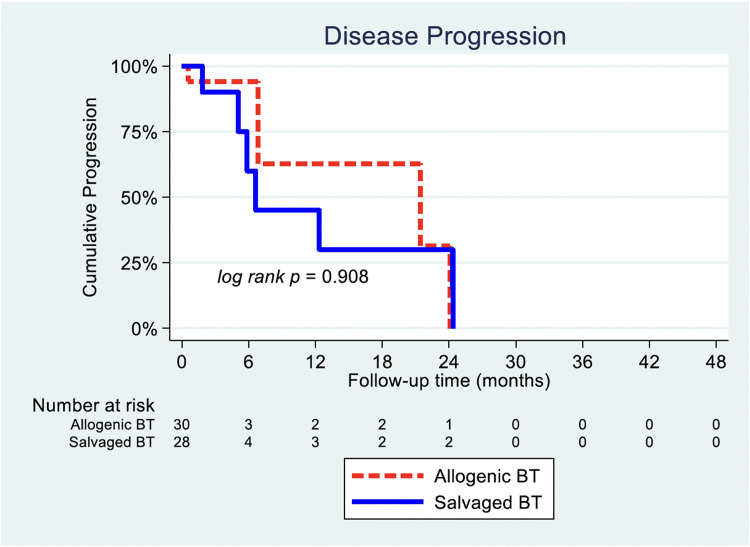
Disease progression: SBT vs. ABT Kaplan–Meier analysis.

The association of SBT and ABT on TP was also analyzed. The crude Kaplan–Meier comparison on disease progression between ABT and SBT PM cohorts showed that the median progression time was 21.4 months for ABT (95% CI 0.1–44.8) and 6.6 months for SBT (95% CI 4.7–8.5), though this was not significant (p=.908, unadjusted HR=1.1, 95% CI 0.29–4.1) (Fig. 3).

**Fig. 4:**
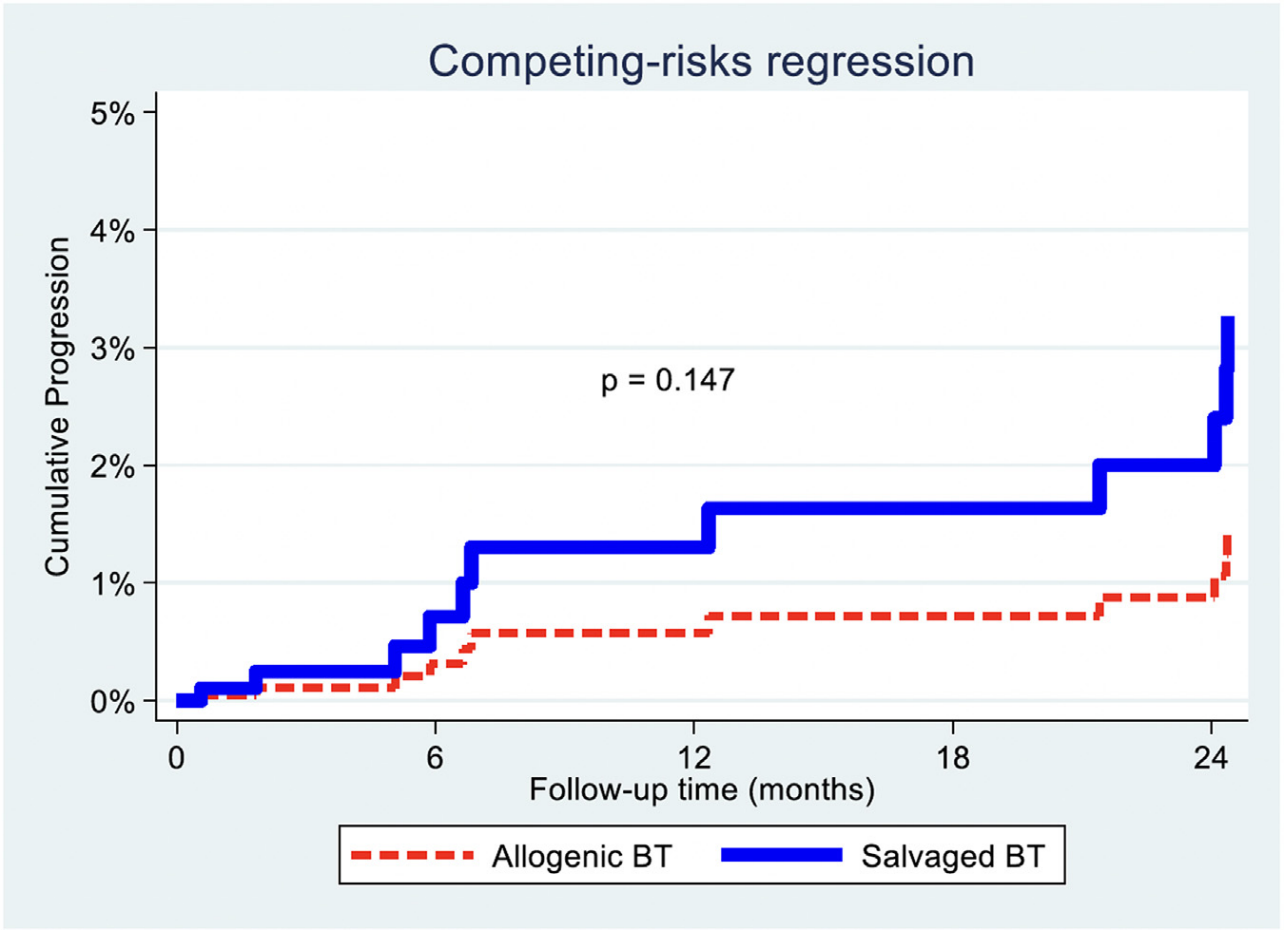
Disease progression with death competing (adjusting for propensity categories).

Our matching distribution table (Table 2) showed that there were insufficient matched disease progression events for a Cox regression with PS-matched analysis, as the Cox shared command was not feasible. Consequently, a death-competing analysis accounting for propensity-matched categories was performed with SBT against ABT, which was again not significant (p=.147, HR=2.29, 95% CI [0.8–7.0]) (Table 5, Fig. 4).

The incidence of both surgical and medical complications were consistent across both matched groups, showing no significant differences. Similarly, the duration of stay in the surgical high dependency unit or intensive care unit, as well as the overall length of hospitalization, were comparable among both PS-matched groups (Table 6).

**Table 6:** Complications according to transfusion types.

Characteristic	Overall (*n*=58)	Blood transfusion (BT) type		
		Allogenic BT (*n*=30)	Salvaged BT (*n*=28)	*Unadjusted* p*-*value
Surgical site-infection (SSI), *n* (%)	2 (3.4)	0 (0.0)	2 (7.1)	.136
Infections other than SSI, *n* (%)	19 (32.8)	12 (40.0)	7 (25.0)	.224
Medical complications, *n* (%)	27 (46.6)	14 (46.7)	13 (46.4)	.986
Surgical site recurrence, *n* (%)	4 (7.4)	1 (3.6)	3 (11.5)	.264
Recurrence requiring operation, *n* (%)	3 (10.0)	1 (5.0)	2 (20.0)	.197
Days of SHDICU stay, median (IQR)	2 (2)	2 (2)	2 (2)	.144
Total length of stay, median (IQR)	22 (34)	23 (32)	22 (33)	.907

## Discussion

PBM strategies have received considerable attention in MSTS [15], with a focus directed toward the implementation of SBT as a pivotal component of PBM. Notably, empirical evidence has underscored the safety profile of SBT [22,31], evincing negligible to absent tumorous cellular presence within salvaged blood subsequent to its passage through a leukocyte depletion filtration system [32]. Preceding retrospective studies have also further elucidated the discernible merits associated with SBT, particularly in its capacity to mitigate the requirement for ABTs, and henceforth, the concomitant increase of overall patient expenditures [33]. In recent years, the usage of SBT has also found considerable application in diverse oncological surgeries such as gastrointestinal and gynecological surgeries [17,34].

Our group has previously established through in vitro studies using flow cytometry that after passage of blood through a cell salvage machine with leucocyte depletion filter, the number of tumor cells in the blood is almost negligible [32]. Moreover, even when tumor cells were detected, the quantity was less than that of the patient’s circulating blood [32], and majority of them are nonviable [35]. This highlights the clinical safety of SBT at cellular level, and serves as the basic foundation toward usage of SBT in MSTS. We have recently published a 2-year clinical outcomes study of our cohort, which showed noninferiority of SBT compared to ABT when analyzing OS and TP outcomes [24]. Our clinical study serves as an in vivo validation from the in vitro results of our previous studies. Despite the above advantages however, there still remains a major reluctance in the uptake of SBT in clinical practice, which possibly is due to the lack of long-term, matched prospective studies comparing the safety profile of SBT against ABT for MSTS patients.

Patients with MSD commonly present with neurological deficits or pain secondary to pathological fracture, which are signs and symptoms of metastatic epidural spinal cord compression. In these situations, most patients require urgent operation, and it is ethically challenging to justify conducting a randomised trial in MSTS patients. Moreover, due to patients having various different primary tumor subgroups, the heterogeneity in their primary tumor profiles results in randomizing to not be feasible. The next best and highest-powered study to be conducted will be a propensity-matched long-term follow-up study, to further substantiate the safety profile of SBT use in MSTS. Our current study fulfils this through PS group matching within patients from the same institution, to reduce the risk of nonsampling errors.

In this present study, we establish how SBT is effective for use in MSTS, with direct PS-matched comparison to ABT. There is no increase in risk of death in MSTS patients or TP, on long-term clinical follow-up. This is especially important due to the fact that blood loss is a significant concern in MSTS [9,10]. In the past, the main method of blood transfusion has been ABT, but various studies have shown an associated increase in patient’s complications due to ABT use [12,14]. In a comprehensive multicenter prospective review encompassing 1601 patients, undertaken by the American College of Surgeons National Quality Improvement Program database [36], 623 individuals (38.9%) underwent red blood cell transfusion. However, recipients of ABT experienced a notably elevated incidence of complications compared to their nontransfused counterparts, with respective rates of 22.3% and 15.0% (p=.00). Hill et al. [37] also found ABT to be an independent risk factor for higher risks of complications while Purvis et al. [14] found that a liberal transfusion policy was associated with an independently higher risk of perioperative morbidity and increasing perioperative costs in MSTS.

This is the first study to prospectively match and compare the survival and TP outcomes in patients undergoing MSTS who received SBT or ABT. There were no significant differences in the oncological characteristics between both PS-matched SBT and ABT groups. Total blood loss and total blood transfusion were also similar (Table 3). There were no significant differences in OS between the PS-matched groups. Although not statistically significant, the point estimates for survival favored the SBT group. From a biological standpoint, this is consistent with existing literature describing the immunosuppressive effects associated with ABT, which may increase susceptibility to postoperative complications such as infections and potentially impact long-term outcomes [24,38]. In contrast, autologous salvaged blood is not known to exert similar immunomodulatory effects. These mechanisms provide biological plausibility for the observed survival estimates, but the present study is not powered to establish a causal or statistically definitive survival advantage.

Our study also did not find any factors that had a significant association with TP. Competing risks analysis was used for TP analysis since PS-matched TP statistical regression did not yield results due to lack of sufficient TP events. Competing risks regression with adjustment for propensity categories showed that there was no significant difference in TP when either ABT or SBT was used. This is likely due to TP being influenced by other factors such as the inherent primary tumor characteristics and ongoing genetic mutations that leads to local and distant progression [39,40].

The comparable trends in OS and TP for the SBT group compared to those observed with ABT after PS matching over a 4-year follow-up period in our study underscores the enduring safety profile of SBT for application in MSTS. In spite of increasing literature debunking the theoretical concerns regarding tumor seeding, the widespread clinical adoption of SBT in MSTS still remains limited today. Within the realm of MSTS, the implementation of PBM strategies emerges as a pivotal approach aimed at enhancing patient outcomes and improving readmission-free survival [41]. Consequently, the integration of SBT as an intraoperative component of PBM initiatives stands to facilitate comprehensive blood loss management, augment outcomes, and reduce overall hospital length of stay durations.

### Limitations

This prospective PS-matched long-term follow-up study is the first to specifically evaluate the long-term safety profile of SBT in patients undergoing MSTS. Nevertheless, several limitations should be acknowledged. The study was nonrandomized due to ethical and practical constraints, as MSTS frequently presents as an urgent condition with neurological compromise, making it neither feasible nor appropriate to impose treatment allocation. Furthermore, the marked heterogeneity of primary tumor biology in MSD would require prohibitively large sample sizes for adequately powered randomised comparisons across individual tumor subtypes. In this context, PS matching represents the most robust and ethically viable methodological approach for comparative evaluation of transfusion strategies in this population.

## Conclusion

SBT has demonstrated considerable promise and utility in intraoperative blood management for patients undergoing MSTS. In our study, we are the first to establish how it exhibits comparable OS and TP outcomes to those observed in patients receiving ABT, even on PS matching.

The integration of SBT into the management of MSTS holds the potential to diminish the need for ABT and its associated risks, consequently enhancing overall patient outcomes in MSTS. Henceforth, there is a compelling case for the widespread adoption of SBT among MSTS surgeons globally, as part of comprehensive PBM protocols.

## Ethical approval

The study was performed in accordance with the ethical standards of the institutional research committee. The National Healthcare Group Domain Specific Review Board (Singapore) has approved the study, with the study reference being 2022/00866.

## Authorship contribution statement

Naresh Kumar: Conceptualization, Writing – review and editing, Supervision, Resources, Funding acquisition. Si Jian Hui: Writing – Original draft, Review and editing, Supervision, Data curation, Project administration. Sheng Liang: Formal analysis. Yong Hao Tan: Data curation, Investigation Balamurugan Vellayappan: Supervision, Methodology. Namith Rangaswamy: Funding acquisition, Writing – review and editing Laranya Kumar: Data duration Youheng Ou Yang: Superivsion, Writing – review and editing James Thomas Patrick Decourcy Hallinan: Project adminisation, Validation, Supervision Jiong Hao Tan: Conceptualization, Writing – review and editing, Supervision, Resources, Funding acquisition.

## Declarations of competing interests

There are no conflicts of interest for the authors of this manuscript.

## Appendix Supplementary materials

Image, application 1
