# Supplementary material for: Shaping the future of blood management in metastatic spine tumor surgery: the case for cell-salvaged transfusion with a propensity-matched study
Source: N Am Spine Soc J. 2026 Feb 5;25:100862. doi: 10.1016/j.xnsj.2026.100862 (PMC12995909; doi:10.1016/j.xnsj.2026.100862)
Supplement: Supplementary file 1 [file mmc1.docx]

**Salvage Blood Publications relevant for changing clinical practice**

# **Papers in ‘International Peer Reviewed Journals**

| **1.** | **‘Paper’** What is the role of autologous blood transfusion in major spine surgery? Naresh Kumar, Chen Y.S., E. Liu, C. Nath. *American Journal Of Orthopaedics,* 2012; 41(6) E89-E95. PMID: 22837998 | **Impact Factor: 1,**  **Citation=21**  **Self Citations:-5** |
| --- | --- | --- |
| **2.** | **‘Paper’** Blood loss in spinal tumour surgery and surgery for metastatic spinal disease: a meta-analysis. Chen Y.S., Tai B.C., D. Nayak, Naresh Kumar, Chua K. H., J.W. Lim, R. Goy, Wong H.K. *Bone Joint J* *2013; 95-B: 683-8.*  *PMID: 23632682 DOI: 10.1302/0301-620X.95B5.31270.* | **Impact Factor: 3.581,**  **Citation:-35**  **Self Citations:-14** |
| **3.** | **‘Paper’** Can There be a Place for Intraoperative Salvaged Blood in Spine Tumor Surgery? Naresh Kumar, Q. Ahmed, Lee V, Chen Y.S., A.S. Zaw, Goy R, Agrawal R. V., Dhewar A. N., Wong H.K. *Ann Surg Oncol 2014 Jul; 21(7): 2436-43.* *Epub 2014 Feb 25.* PMID: 24566859 *DOI: 10.1245/s10434-014-3569-x.* | **Impact Factor: 3.857,**  **Citation:-15**  **Self Citations: 7** |
| **4.** | **‘Paper’** Use of intraoperative cell-salvage for autologous blood transfusions in metastatic spine tumour surgery: a systematic review. Naresh Kumar, Chen Y.S., A.S. Zaw, D. Nayak, Q. Ahmed, Soong R, Wong H.K. *Lancet Oncology, 2014; 15(1): e33-41.* PMID: 24384492 *DOI:10.1016/S1470-2045(13)70245-6.* | **Impact Factor: 36.418,**  **Citation=43**  **Self Citations: 8** |
| **5.** | **‘Paper’** Flow Cytometric Evaluation of the Safety of Intraoperative Salvaged Blood Filtered with Leucocyte Depletion Filter in Spine Tumour Surgery – Naresh Kumar, R. Lam, A.S. Zaw, R. Malhotra, Tan J.H. Jonathan, G. Tan, T. Setiobudi. *Ann Surg Oncol, 2014 Dec; 21(13): 4330-5. Epub 2014 Jul 29.* PMID:25069862 *DOI:10.1245/s10434-014-3950-9.* | **Impact Factor: 3.857,**  **Citation:-13**  **Self Citations:-6** |
| **6.** | **‘Paper’** Are we ready for the use of intraoperative salvaged blood in metastatic spine tumour surgery? Kumar, Naresh, Ahmed, Qasim, [Lee, Victor K. M.](https://meprd.nus.edu.sg/userprofile.html?uid=1756), Zaw, Aye Sandar, Goy, Raymond, [Wong, Hee Kit](https://meprd.nus.edu.sg/userprofile.html?uid=184). [*European Spine Journal*](https://link.springer.com/journal/586) *19 Jul 2015.* *25, pages3997–4007* *DOI: 10.1007/s00586-015-4112-x* |  |
| **7.** | **‘Paper’** Blood Loss and Transfusion Requirements in Metastatic Spinal Tumor Surgery: Evaluation of Influencing Factors. Naresh Kumar, A.S. Zaw, Khine H.E., K. Maharajan, Wai K.L., Tan W.L. Barry, S. Mastura, R. Goy. [Ann Surg Oncol.](http://www.ncbi.nlm.nih.gov/pubmed/?term=Blood+loss+and+transfusion+requirements+in+Metastatic+Spine+Tumour+Surgery)*2016 Jun;23(6):2079-86* *PMID: 26819232*  DOI:[10.1245/s10434-016-5092-8](https://doi.org/10.1245/s10434-016-5092-8) | **Impact Factor:3.857**  **Citation:-17**  **Self Citations:-8** |
| **8.** | **‘Paper’** Are We Ready for Use of Salvaged Blood in Metastatic Spine Tumour Surgery? Naresh Kumar, Q. Ahmed, V. Lee, A.S. Zaw, R. Goy, Wong H.K. *EurSpine J. 2016*. *Dec;25(12):3997-4007.*  *PMID: 26188770 DOI:10.1007/s00586-015-4112-x* | **Impact Factor:2.066**  **Citation:-10**  **Self Citations:-7** |
| **9.** | **‘Paper’** Intraoperative cell salvage in metastatic spine tumour surgery reduces potential for reinfusion of viable cancer cells. Naresh Kumar, A.S. Zaw, B.L. Khoo, S. Nandi, Z.Lai, G. Singh, C.T. Lim, J.P. Thiery. *EurSpine J. 2016*. *Dec;25(12):4008-4015.* PMID: 26951173 *DOI: 10.1007/s00586-016-4478-4.* | **Impact Factor:2.066**  **Citation:-12**  **Self Citations:-4** |
| **10.** | **‘Paper’** Is Autologous Salvaged Blood a Viable Option for Patient Blood Management in Oncologic Surgery? – AS. Zaw, SB Kantharajanna, Naresh Kumar. *Transfus Med Rev. 2017 Jan;31(1):56-61.* PMID:27421661*DOI: 10.1016/j.tmrv.2016.06.003* | **Impact Factor: 4.111**  **Citation:-9**  **Self Citations:-2** |
| **11.** | **‘Paper’ ­**Perioperative Blood Transfusion: Does It Influence Survival and Cancer Progression in Metastatic Spine Tumor Surgery? AS. Zaw, Kantharajanna SB, Maharajan K, Tan B, Vellayappan B, Naresh Kumar. *Transfusion 2017 Feb*; 57(2):440-450*. PMID: 27828812 DOI: 10.1111/trf.13912* | **Impact Factor: 3.423**  **Citation:-9**  **Self Citations:-3** |
| **12.** | **‘Paper’** [Metastatic spine tumor surgery: does perioperative blood transfusion influence postoperative complications?](https://meprd.nus.edu.sg/viewobject.html?cid=1&id=1686340) Zaw AS, Kantharajanna SB, Maharajan K, Tan B, Saparamadu AA, Kumar N. *Transfusion, 16 September 2017.* 57: 2790-2798. DOI: 10.1111/trf.14311 |  |
| **13.** | **‘Paper’** Metastatic Efficiency of Tumour Cells Can be Impaired by Intraoperative Cell Salvage Process: Truth or Conjecture? Naresh Kumar, A.S. Zaw, Shashidhar B, B.L. Khoo, C.T. Lim, J.P. Thiery. *Transfusion Medicine 2017*. Volume 27(5), October 2017: 327–334 PMID:28833768 DOI:[10.1111/tme.12453](https://doi.org/10.1111/tme.12453) | **Impact Factor:1.798**  **Citation:-4**  **Self Citations:-1** |
| **14.** | **‘Paper’** - Metastatic Spine Tumour Surgery: Does Perioperative Blood Transfusion Influence Postoperative Complications? Zaw AS, Shashidhar BK, Maharajan K, B Tan, A Sapramadu, Naresh Kumar. *Transfusion 2017.* Volume 57(11), November 2017; 2790–2798 PMID: 28921529 DOI: 10.1111/trf.14311 | **Impact Factor:3.423**  **Citation:-2**  **Self Citations:-1** |
| **15.** | **‘Invited Article’** Current Status of Use of Salvaged Blood in Metastatic Spine Tumour Surgery (MSTS). Naresh Kumar, Nivetha R, JYH Tan, K Akbary, RS Patel, R Kannan, *Neurospine*. 2018 Sep;15(3):206-215. PMCID: PMC6226127 PMID: 30071572 DOI: [10.14245/ns.1836140.070](https://doi.org/10.14245/ns.1836140.070) | **Impact Factor:-**  **Citation:-** |
| **16.** | **‘Paper’** Evaluation of the Feasibility of Transfusing Leucocyte Depletion Filter (LDF) Processed Intra-Operative Cell Salvage (IOCS) Blood in Metastatic Spine Tumour Surgery (MSTS): Protocol for a Non Randomised study (Preprint ahead of publication). Kumar, Naresh, Tan, Jiong Hao, Ravikumar, Nivetha, Tan, Joel Yong Hao, Milavec, Helena, Agrawal, Rohit, Kannan, Rajesh, Kumar, Aravind. *JMIR Research Protocols.* 11 Nov 2019. DOI: http://dx.doi.org/10.2196/16986 | **Impact Factor: 1.1**  **Citation:-**  **Self Citations:-** |
| **17.** | **‘Paper’** Readmission-free survival analysis in metastatic spine tumour surgical patients: A novel concept. Naresh Kumar, A Thomas, M Ramos, S Madhu, JYH Tan, L Shen, K Lopez, A Villanueva, J Tan, B Vellayappan. *Annals of Surgical Oncology.*(2021). DOI: 10.1245/s10434-020-09404-7 | **Impact Factor: 3.780**  **Citation:-**  **Self Citations:-** |
| **18.** | **‘Paper’** Intraoperative cell-salvaged autologous blood transfusion is safe in metastatic spine tumour surgery: early outcomes of prospective clinical study. Naresh Kumar, Joel Yong Hao Tan, Zhaojin Chen, Nivetha Ravikumar, Helena Milavec & Jiong Hao Tan. *Eur Spine J 32, 2493-2502. 2023 May 16. DOI:* 10.1007/s00586-023-07768-4. PMID: 37191676 | **Impact Factor:2.634**  **Citation:-**  **Self Citations:-** |
| **19.** | **‘Paper’** Does patient blood management affect outcomes in metastatic spine tumour surgery? A review of current concepts. Kumar N, Lee, Eunice Xin Yi, Hui, Si Jian, Kumar, Laranya., Tan, Jonathan Ashokka, Balakrishnan. *GSJ. 2023.*  DOI: 10.1177/21925682231167096 | **Impact Factor: 2.915**  **Citation:-**  **Self Citations:-** |

#

# **Research Grant Awarded**

| **1.** | **‘NMRC Grant’** received the **NMRC-NIG-1055-2011** grant for the study titled – ‘Is it safe to transfuse Leucocyte Depletion Filter (LDF) processed Intra-Operative Cell Salvage (IOCS) blood, in spine tumour surgery?’ Nov 2010 | **Approved: Value of SGD 200,000** |
| --- | --- | --- |
| **2.** | **‘2014 AOSpine East Asia Research Award’** received AOSpine grant **AOSEA(R)2014-01** to collect preliminary data using flow cytometry to answer “Is it safe to transfuse Leucocyte Depletion Filter (LDF) processed Intra-Operative Cell Salvage (IOCS) blood, in spine tumour surgery?” Feb 2014 | **Approved: Value of SGD 7,500(CHF7,000)** |
| **3.** | **‘NUHS Bridging Funds 02/FY13’** received NUH Bridging Grant till we get the clinical trial grant – to extend our study ‘Is it safe to transfuse Leucocyte Depletion Filter (LDF) processed Intra-Operative Cell Salvage (IOCS) blood in spine tumour surgery?’ to whole `musculoskeletal tumour surgery. Apr 2014 | **Approved: Value of SGD 75,000** |
| **4.** | **‘2015 AOSpine East Asia Research Award’** received AOSpine grant **AOSEA(R)2015-03 t**o conduct a study to establish that tumour cell count in the blood salvaged with cell saver during metastatic spine tumour surgery is significantly less than that in the circulation of the patient. Jun 2015 | **Approved: Value of SGD 7,500(CHF7,000)** |
| **5.** | **‘MOE Academic Research Fund (AcRF) Tier 1 Grant’** received for the project ‘Novel Tumor Cell Filter System for Intra-operative Blood Salvage Autotransfusion’. Jun 2015 | **Approved: Value of SGD 138,000** |
| **6.** | **‘NMRC-NIG Grant’** received a **– NMRC/CNIG/1161/2016** for ‘A Prospective Study to Evaluate the Safety and Efficacy of Transfusing Intraoperative Salvaged Blood in Metastatic Musculoskeletal Tumour Surgery. As a PI Mentor | **Approved: Value of SGD 150,000** |

**Innovative Ideas and Research causing a Paradigm Shift in Spine Surgery, Orthopaedic Surgery & Orthopaedic Education**

| **1.** | **Conceptualisation to Bedside Implementation of Salvaged Blood in Tumour Surgery:**  First ever academic surgeon to **conceptualise the use of salvaged blood** in metastatic spine tumour surgery and **validate its safe** **use** in metastatic spine tumour surgery through well-structured experimental procedures. The evolution of this thought process can be clearly understood through the two publications namely:   1. Use of intraoperative cell-salvage for autologous blood transfusions in metastatic spine tumour surgery: a systematic review. Naresh Kumar et al. *Lancet Oncology, 2014; 15: e33-41.* PMID: 24384492 *DOI: 10.1016/S1470-2045(13)70245-6.* |
| --- | --- |
